# Differential Gene Expression Caused by the *F* and *M* Loci Provides Insight Into Ethylene-Mediated Female Flower Differentiation in Cucumber

**DOI:** 10.3389/fpls.2018.01091

**Published:** 2018-08-14

**Authors:** Jian Pan, Gang Wang, Haifan Wen, Hui Du, Hongli Lian, Huanle He, Junsong Pan, Run Cai

**Affiliations:** School of Agriculture and Biology, Shanghai Jiao Tong University, Shanghai, China

**Keywords:** cucumber, ethylene response, sex differentiation, unisexual flower, floral development

## Abstract

In cucumber (*Cucumis sativus* L.), the differentiation and development of female flowers are important processes that directly affect the fruit yield and quality. Sex differentiation is mainly controlled by three ethylene synthase genes, *F* (*CsACS1G*), *M* (*CsACS2*), and *A* (*CsACS11*). Thus, ethylene plays a key role in the sex differentiation in cucumber. The “one-hormone hypothesis” posits that *F* and *M* regulate the ethylene levels and initiate female flower development in cucumber. Nonetheless, the precise molecular mechanism of this process remains elusive. To investigate the mechanism by which *F* and *M* regulate the sex phenotype, three cucumber near-isogenic lines, namely H34 (*FFmmAA*, hermaphroditic), G12 (*FFMMAA*, gynoecious), and M12 (*ffMMAA*, monoecious), with different *F* and *M* loci were generated. The transcriptomic analysis of the apical shoots revealed that the expression of the B-class floral homeotic genes, *CsPI* (*Csa4G358770*) and *CsAP3* (*Csa3G865440*), was immensely suppressed in G12 (100% female flowers) but highly expressed in M12 (∼90% male flowers). In contrast, *CAG2* (*Csa1G467100*), which is an AG-like C-class floral homeotic gene, was specifically highly expressed in G12. Thus, the initiation of female flowers is likely to be caused by the downregulation of B-class and upregulation of C-class genes by ethylene production in the floral primordium. Additionally, *CsERF31*, which was highly expressed in G12, showed temporal and spatial expression patterns similar to those of *M* and responded to the ethylene-related chemical treatments. The biochemical experiments further demonstrated that *CsERF31* could directly bind the promoter of *M* and promote its expression. Thus, *CsERF31* responded to the ethylene signal derived from *F* and mediated the positive feedback regulation of ethylene by activating *M* expression, which offers an extended “one-hormone hypothesis” of sex differentiation in cucumber.

## Introduction

Cucumber (*Cucumis sativus* L.), a horticultural crop consumed worldwide, has the third highest production quantity (61 million tons in 2016) after tomato and onion, and China leads in the production of cucumber with 76% of all production (Un Food and Agriculture Organization Corporate Statistical Database [Faostat], 2017). The cucumber is more than an agronomically important vegetable, and due to the diversity of its flower sexual types and plant sexual systems (flower sexual types that express on a single plant), the cucumber is an ideal model for investigating the mechanism of sex differentiation in unisex flowers (Malepszy and Niemirowicz-Szczytt, 1991). The sexual types of the cucumber flower include the female flower, male flower, and bisexual flower. According to these sexual types, the following five cucumber sexual systems have been categorized: monoecy (presence of female and male flowers), gynoecy (only female flowers), androecy (only male flowers), andromonoecy (male and bisexual flowers), and hermaphrodite (only bisexual flowers) (Galun, 1962; Kubicki, 1969a,b,c; Fruchard and Marais, 2017). Notably, the variety seen in the cucumber sexual systems is rare in the plant kingdom, covering ∼95% of sexual systems among angiosperms (Käfer et al., 2017). Treatment with exogenous ethylene or ethylene-related chemicals can affect the sex of the flowers in cucumber plants (MacMurray and Miller, 1968; Iwahori et al., 1969). The ethylene-mediated differentiation of unisexual flowers in cucumber has been explained by the “one-hormone hypothesis" (Yin and Quinn, 1995). This hypothesis posits that ethylene in cucumber inhibits maleness and induces femaleness by modulating the expression levels of *F* and *M* (Yin and Quinn, 1995; Trebitsh et al., 1997). According to this hypothesis, the *F* locus encodes a gene for ethylene production, and the *M* locus encodes an ethylene-response factor that might be an ethylene receptor. This hypothesis has been modified and improved based on the map-based cloning of *M*, and an *M*-mediated positive feedback regulatory mechanism of ethylene has been proposed (Li et al., 2008, 2009, 2012). In our previous study, sex expression was associated with the fruit shape (Tan et al., 2015). In most *M* mutant lines (*mm* genotype), the gynoecium presents shorter ovaries and oval/round fruit, whereas the *MM* genotype presents elongated ovaries and normal long fruit, suggesting that *M* promotes an ovary of better quality via a positive feedback mechanism. The expression analysis also demonstrated that the *M* transcript began to accumulate beneath the pistil primordia of the flower buds during the bisexual stage (Saito et al., 2007).

Many studies have attempted to identify the “sex genes” in cucumber, and three Mendelian loci – *F/f*, *M/m*, and *A/a*, have been identified to be responsible for the different sexual systems (Pierce and Wehner, 1990). Using map-based cloning, the “sex genes” (*F*, *M*, and *A*) have been cloned from the aforementioned loci (Mibus and Tatlioglu, 2004; Li et al., 2009; Boualem et al., 2015). Unsurprisingly, *F*(*CsACS1G*), *M*(*CsACS2*), and *A*(*CsACS11*) all encode 1-aminocyclopropane-1-carboxylate synthase (ACS), which catalyzes the rate-limiting step in ethylene biosynthesis; the association between ACS and ethylene has been known for decades.

The melon (*C. melo*) serves as another well-known model in sex differentiation studies. In the melon, the sex-determination pathway model integrating *CmACS11* (i.e., *A* in both cucumber and melon), *CmWIP1* (i.e., *G* in melon and *CsWIP1* in cucumber), and *CmACS7* (i.e., *M* in both melon and cucumber) explains the primary mechanism of male, female, and bisexual flower generation (Boualem et al., 2015). This model can be similarly applied to the cucumber according to the current study. Briefly, female flowers are initiated due to the expression of *A*, which represses the expression of *CsWIP1*. Thus, the non-expression of *CsWIP1* promotes the expression of *M*, which inhibits stamen development. If a non-functional *M* is expressed, hermaphroditic flowers develop instead of female flowers. The loss of function of *A* generates androecious plants, leading to the expression of *CsWIP1* at the whole plant level. Notably, *F*, as a gain-of-function structural variation (Zhang et al., 2015), was not mentioned in this model. However, these findings have triggered further questions regarding the mechanism by which ethylene (produced by *F*, *M*, and *A*) controls sex expression; furthermore, the mechanism by which the *ACS* genes were recruited to form a regulatory mechanism in cucumber female flower evolution remains unclear.

In addition to *F*, *M*, and *A*, other ethylene biosynthetic genes, such as *CsACO2* and *CsACO3*, which encode ACC oxidases, are involved in sex expression. However, the transcript levels of *CsACO2* and *CsACO3* in the shoot apices are inversely correlated with femaleness, indicating the potential presence of feedback inhibition that controls ethylene production (Kahana et al., 1999). In addition, *CsACO2* can influence floral organ development in both cucumbers and *Arabidopsis* (Duan et al., 2008; Chen et al., 2016). *CsWIP1* can directly bind the promoter of *CsACO2* to repress its expression, suggesting that *CsWIP1* plays the same key role as *G* (*CmWIP1*) in sex differentiation (Chen et al., 2016). An ethylene receptor gene, *CsETR1*, has been shown to participate in stamen arrest via the induction of DNA damage (Wang et al., 2010). Further studies suggest that a cucumber nuclease-encoding gene, *CsCaN*, responds to the ethylene signal and allows the DNA of the stamen primordia to damage the developing female flowers (Gu et al., 2011).

Previous studies have provided information and theories suggesting that hormones, particularly ethylene, promote femaleness in cucumbers. However, molecular and biochemical data are scarce, limiting our understanding of the sex determination mechanism in cucumber and preventing our ability to artificially control the quantity and quality of female flowers. To investigate the ethylene-mediated sex expression process and the interplay mechanism between *F* and *M*, three cucumber near-isogenic lines (NILs), namely H34, G12, and M12, with different *F* and *M* loci were produced by backcrossing. We found that several novel B- and C-class floral homeotic genes were differentially expressed between the NILs by comparing the transcriptomes of the shoot apices. ERF transcription factors, which act as transacting regulators in the final step of the ethylene signaling pathway, bind to conserved elements, such as the GCC-box or ERE-box, in the promoters of many ethylene-inducible genes (Ohme-Takagi and Shinshi, 1995; Solano et al., 1998; Han et al., 2016). An ERF family gene, *CsERF31*, was identified among the differentially expressed genes (DEGs) because its spatiotemporal expression correlated with *M*. Furthermore, we demonstrated that *CsERF31* could directly bind the promoter of *M* to promote its expression. In this study, we propose an extended “one-hormone hypothesis” of sex differentiation and development of cucumber.

## Materials and Methods

### Plant Materials and Growth Conditions

**FIGURE 1:**
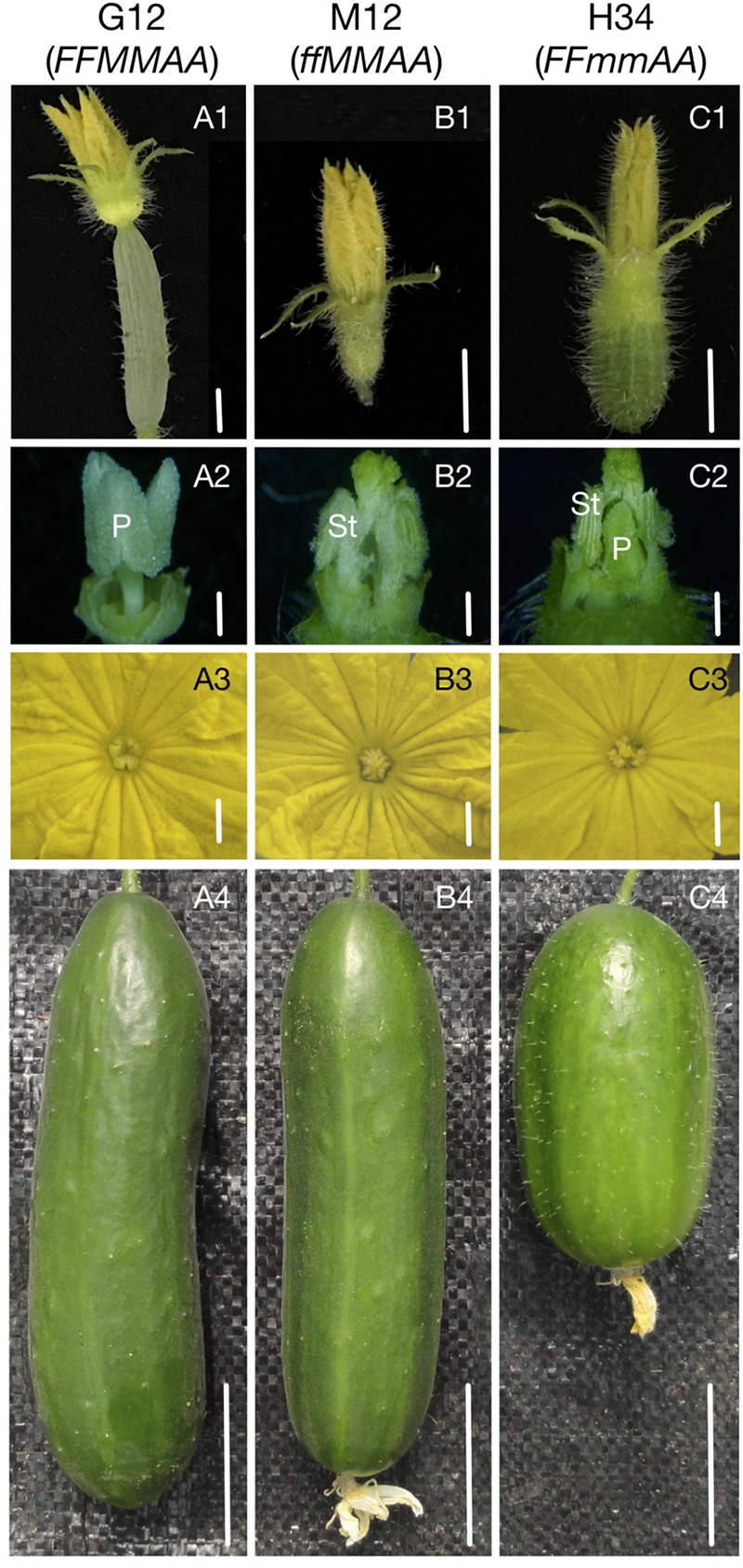
Sex expression, flower, and fruit morphology of NILs. Flower organs showed different morphology between G12 (*FFMMAA*), M12 (*ffMMAA*), and H34 (*FFmmAA*). **(A1–C1)** Flowers before antheses. Bars = 0.5 cm, **(A2–C2)** Floral organ without petal and sepal. Bars = 0.2 cm, **(A3–C3)** Floral organ with petal. Bars = 0.2 cm, **(A4–C4)** Mature fruit. Bars = 4 cm. P, pistil; St, stamen.

To explore the genes and gene networks that control the sex expression in cucumbers, we performed RNA-Seq analyses of shoot apices from three NILs – G12 (*FFMMAA*), M12 (*ffMMAA*), and H34 (*FFmmAA*). The G12 was substituted for *M* loci from H34 via seven generations of backcrosses by our marker-assisted selection program (Li et al., 2009). The M12 was a spontaneous mutant from G12 with a loss of *F* (*CsACS1G*), and has been stabilized via five generations of self-crosses. The NILs belong to the European greenhouse cultivar and have short gloss fruits covered with small spines without warts (**Figures 1A4–C4**). No visible difference was observed among the NILs in terms of the plant morphology, except that G12 and H34 express female and bisexual flowers, respectively, at the whole plant level, while M12 bears only male flowers before the 25th ± 3 node in the main-stem (**Figure 1B4**); however, the female flowers can be observed in the lateral branches. As the *F* and *M* loci cause significant differences prior to the morphological changes, we chose shoot apices that contained 1- to 5-mm buds for the transcriptome analyses.

The seeds of the NILs were germinated on a wet filter paper in a Petri dish at 28°C in the dark overnight. This was followed by growing the resulting seedlings in an artificial climate room for 16 h/8 h at 25°C/18°C during the day/night. The samples used for the gene expression analysis were collected from the plants in the artificial climate room. The chemically treated and control plants were transferred to a greenhouse at the Shanghai Jiao Tong University in April 2017.

### Preparation of RNA Samples for RNA-Seq and Quantitative Real-Time RT-PCR (qRT-PCR)

The shoot apices from the aforementioned cucumber plants were collected at the four-leaf stage. The samples were immediately frozen in liquid nitrogen and stored at −80°C. The total RNA was isolated using an RNA extraction kit (Epigenetics, United States). The RNA was checked for contamination using RNase-free agarose gel electrophoresis, and then, the RNA purity was examined using a NanoDrop Spectrophotometer (THERMO, United States). The RNA integrity was measured and assessed using an Agilent 2100 Bioanalyzer system (Agilent, United States).

For the qRT-PCR experiment, G12 flower buds with different lengths of 1, 2, 5, and 10 mm were collected. A day before flowering, the petal, stamen, stigma, mesocarp, and exocarp were isolated from the G12 female flowers. Shoot apices treated with chemicals were isolated from the chemically treated and control plants as described below.

### Transcriptome Library Construction and Sequencing

The RNA-Seq for comparative transcriptomic analyses of three genotypes were performed with three biological replicates. The library construction and sequencing were performed using a BGISEQ-500 by Beijing Genomic Institution (BGI, China). The genomic DNA was removed with two digestions using Amplification grade DNase I (Epigenetics, United States). The RNA was sheared and reverse transcribed using random primers to obtain the cDNA, which was used for the library construction. The library quality was determined using a Bioanalyzer 2100 (Agilent), and then the library was used for sequencing using the sequencing platform BGISEQ-500 (BGI, China) (Fehlmann et al., 2016). All the generated raw sequencing reads were filtered to remove reads with adaptors and reads in which unknown bases were greater than 10% of low-quality reads. The clean reads were obtained and saved in the FASTQ format. The clean data have been uploaded to the European Nucleotide Archive (ENA) (Project ID: PRJEB25272, Sample ID: SAMEA104656171, SAMEA104656172, SAMEA104656173, SAMEA104656174, SAMEA104656175, SAMEA104656176, SAMEA3469513, SAMEA3469514, SAMEA3469515).

### Bioinformatics Analysis of RNA-Seq Data

We used Bowtie2 (Langmead et al., 2009) to map the clean reads to the reference genome, (Cucumber_ChineseLong_v2^1^, Huang et al., 2009). The read counts were summarized, and the Fragments Per Kilobase of exon per Million fragments mapped (FPKM) was calculated for each annotation on the reference sequence. The NOISeq method (Tarazona et al., 2011) was used to screen for DEGs between the groups. In this study, an absolute value of log2foldchange ≥ 0.6 and diverge probability ≥ 0.6 were used as cut-offs to screen for gradual expression differences among the three NILs (**Supplementary Tables S2**, **S3**).

A Venn diagram analysis was performed using Venny tools (Oliveros, 2007–2015). An expression trend analysis was performed using OmicShare^2^ tools. To identify the homolog in *Arabidopsis*, we used the cucumber protein IDs (Huang et al., 2009) to batch query the *Arabidopsis* proteins (TAIR10) using BLASTP on the Cucurbit Genomics Database^1^ with an e-value cut-off of 1e^−1^. Based on the cucumber homolog in *Arabidopsis*, the protein interaction network was predicted using STRING^3^, which is a database that aims to provide a critical assessment and integration of protein–protein interactions, including direct (physical) and indirect (functional) associations. The prediction of protein–protein interaction was measured by the combined score, which was computed by combining the probabilities from the different evidence channels and corrected for the probability of randomly observing an interaction.

### Chemical Treatments

The ethylene-related chemicals used in this study included ethephon (an ethylene-releasing agent), aminoethoxy vinyl glycine (AVG, ethylene biosynthesis inhibitor), and AgNO_3_ (ethylene action inhibitor). These three chemicals were purchased from Sigma (China). All cucumber plant treatments were performed according to our previous studies (Li et al., 2012). Each treatment employed ten plants, and five plant shoot apices were excised for RNA extraction after the treatment. To observe the effects of the chemical application on the sexual expression of cucumber plants, the other five plants of each treated line were allowed to grow to maturity. The sexual types were recorded up to the 25th node on the main stem.

### Gene Expression Analyses by qRT-PCR

The cDNA used for the qRT-PCR was reverse transcribed from 1 mg of total RNA using a QuantScript RT Kit (TIANGEN, China). The qRT-PCR analyses were carried out using FastStart Essential DNA Green Master (Roche, United States) on a Rotor-Gene Q Real-Time System (QIAGEN, United States). The gene-specific primers used for the qRT-PCR were designed using Primer 3 software. The cucumber *actin* was used as an internal control to normalize the expression data (Li et al., 2012). Five biological replicates for chemical treatments and three biological replicates for other samples were used per gene. Each qRT-PCR experiment was performed with three technical replicates. The gene-specific primers are listed in **Supplementary Table S5**.

All data were expressed as the mean values ± standard deviation (SD) of biological replicates. Differences were analyzed with one-way ANOVA using SAS 9.1.3 software. The *P*-values < 0.05 were considered to be significant.

### Yeast One-Hybrid Assays

For the yeast one-hybrid assay, the open reading frames (ORFs) of *CsERF31* were amplified using PrimerStar GXL DNA polymerase (TAKARA, Japan) and then cloned into pB42AD (EcoRI/XhoI) using a ClonExpress II One Step Cloning Kit (Vazyme, China). The two ERE-boxes in the *M* promoter (18 bp length containing the ERE-box element, with 3 tandem repeats) were inserted into the pLacZ vector (**Figure 5C**). The analyses were performed using the yeast EGY48a strain as previously described, and empty vectors were used as negative controls. The transformants were cultivated on SD/-Leu/-Ura medium and tested on the SD/-Leu/-Ura medium with X-gal (5-Bromo-4-chloro-3-indolyl β-D-galactopyranoside) (Yan et al., 2017).

### Dual-Luciferase (Dual-LUC) Assay

This assay was performed as previously described (Luo et al., 2014). Briefly, the ORFs of *CsERF31* were cloned into the pHB vector to be driven by a 35S promoter for over-expression. This was followed by the recombinant vectors being separately transformed into *Agrobacterium tumefaciens* GV3101 as effectors. The 2 and 1-kb promoters of *M* were cloned into the pGREEN0800 vector to drive the firefly *luciferase* (*LUC*) reporter gene. Each *M* promoter vector was then co-transformed with the helper plasmid pSoup19 into *Agrobacterium* GV3101 as reporters. The reporter and effector were mixed at a 1:2 volume ratio and injected into tobacco (*Nicotiana benthamiana*) leaves. An empty pHB vector was used as a negative control. The constitutive 35S promoter driving *Renilla luciferase* (*REN*) was used as an internal reference. The leaf samples used in the Dual-LUC assay were collected after 2 days using commercial Dual-Luciferase reaction reagents according to the manufacturer’s instructions (Promega, United States). Four biological replicates of each sample were measured using a GloMax 20/20 Luminometer (Promega, United States). The primers used in the yeast one-hybrid and Dual-LUC assay are listed in **Supplementary Table S5**.

## Results

### Comparison of Gene Expression Among Three NILs With Different Genotypes

The RNA-Seq sequencing generated ∼24 million single-end reads for each sample, and three biological replicates were performed for each line (**Supplementary Table S1**). By performing a bioinformatics analysis of the RNA-Seq data, we identified 631 DEGs (**Supplementary Table S2**), including 281 genes that were upregulated and 350 genes that were downregulated in G12 compared to those in H34; in addition, we identified 534 DEGs (**Supplementary Table S3**), including 327 genes that were upregulated and 207 genes that were downregulated in G12 compared to those in M12.

To verify the DEGs identified by RNA-Seq, we performed qRT-PCR assays using independently collected samples that were at the same developmental stage as those used for the RNA-Seq analysis. A total of 20 predicted sex differentiation-related genes were selected from the DEGs. All the 20 genes showed the same expression patterns in the qRT-PCR assays as the RNA-Seq data (**Figure 3** and **Supplementary Figure S1**). The Pearson’s correlation coefficients between the qRT-PCR and RNA-Seq data were H34 = 0.92904 and M12 = 0.99016 (*P* < 0.001) when compared with G12, respectively, indicating that the RNA-Seq data were highly reliable.

### Phytohormone and Floral Regulators Co-regulate With *M* in Ethylene-Mediated Female Development

**FIGURE 2:**
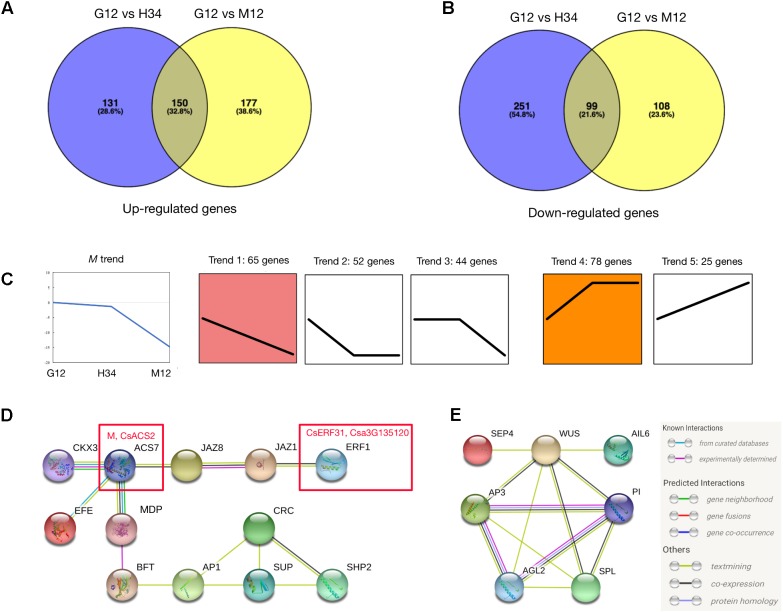
Analysis to screening *M*-related genes from DEGs. Venn diagrams of DEGs that were upregulated **(A)** or downregulated **(B)** between different genotypes. Expression trend analysis of DEGs was performed to screen genes that have downregulated expression trends (Trend 1, 2, and 3) similar to *M* (**C**, left) and upregulated trends (Trend 4 and 5) that are opposite. A total of 264 genes were classified into 5 trends and the best homolog was identified in *Arabidopsis* **(C)** (**Supplementary Table S4**). The predicted protein interaction network **(D)** showed the homologs that were associated with ACS7, homolog of M (**D**, left red box). The interaction network **(E)** showed the homologs that were associated with B-class floral protein (AP3 and PI) and shared opposite expression trend with *M*. In the network, the links between proteins signify the various interaction data supporting the network, colored by evidence type (**E**, right inset). Information of genes presented in **(D)** and **(E)** are listed in **Table 1**.

We performed a series of analyses to screen for *M*-related genes among the DEGs. Since *M* was activated by the ethylene produced by *F* (Li et al., 2012), the DEGs between G12 and M12 contained genes which activated *M* and were involved in the *M* positive-feedback. However, the DEGs between G12 and H34 only contained genes which were involved in the *M* positive-feedback, because *M* (in G12) and *m* (a loss-of-function M, in H34) are both activated by *F* but *m* could not initiate the positive-feedback. A total of 264 genes filtered by the Venn and Expression trend analyses were selected (**Figures 2A–C** and **Supplementary Table S4**), and the genes that were most homologous to *Arabidopsis* genes were identified. We then performed a STRING analysis (Szklarczyk et al., 2015) to predict the association networks of the homologs. The network showed that 11 homologs that were associated with ACS7 (homolog of M) (**Figure 2D**) and 7 homologs which had the opposite expression trends with *M* were related with floral organs development (**Figure 2E**).

In the predicted protein interaction network, several phytohormone and floral regulators were linked to *ACS7*. For example, JAZ1 and JAZ8 have a protein-protein interaction (Chini et al., 2009) and regulate ERF1 via EIN3/EIL1 in *Arabidopsis* (Song et al., 2014) (**Figure 2D**). Accordingly, *SUP*, which is a well-known negative transcriptional regulator of B-class floral genes, and SHP2 (i.e., CAG2, AG-like C-class floral gene) were indirectly linked to *ACS7*. Notably, *ERF1*, which is a homolog of *CsERF31*, belongs to the EREBP (ethylene-response element binding protein) subfamily, which mediates the response to ethylene, suggesting that *CsERF1* may play a key role in the ethylene response pathway of *F* and *M* (**Figure 2D**). Among the homologs which had the opposite expression trends with *M*, several well-study genes, such as AP3 (*CsAP3*), WUS (*CsWUS*), and SPL (*CsSPL*) were screened out, suggesting that these genes had interactions with B-class genes during floral developments in cucumber (**Figure 2E**).

### *CsERF31* and *M* Had Similar Expression Patterns and Responses to the Ethylene-Related Chemical Treatments

**FIGURE 3:**
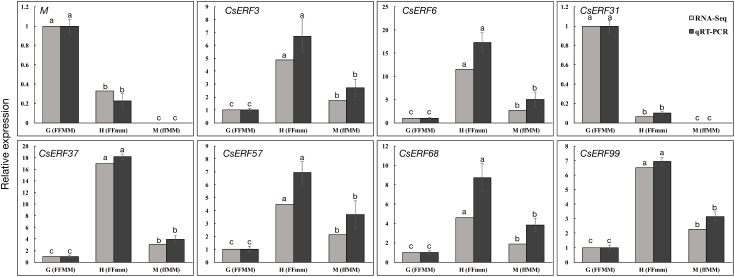
qRT-PCR validation of ERF family genes identified by RNA-Seq. Seven ERF family genes and *M* were selected from DEGs of the two sets of transcriptome comparisons (G12 vs. M12, and G12 vs. H34) for qRT-PCR confirmation. The cucumber *Actin* was used as an internal control (Li et al., 2012), and these experiments were repeated with three biological samples. Error bars represent the SD from three biological replicates. Different letters (a–c) indicate significant differences (*P* < 0.05) of 7 ERF family genes and *M* in G12, H34, and M12.

**FIGURE 4:**
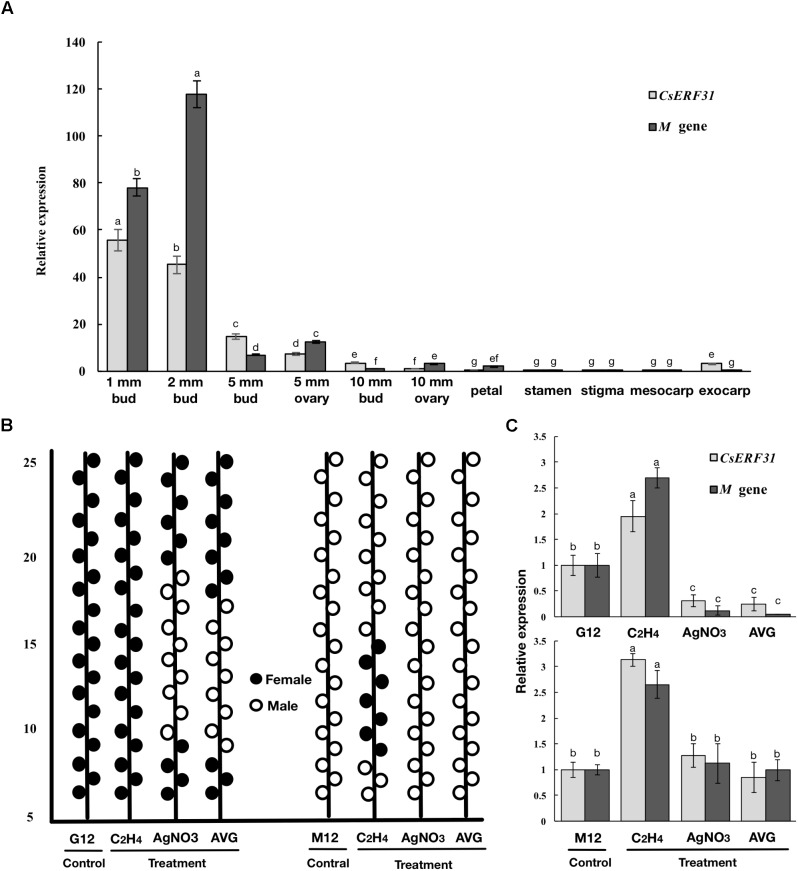
Expression patterns of *M* and *CsERF31* in flower organs and shoot apices treated by ethylene-related chemical. **(A)** Temporal expression patterns of *M* and *CsERF31* at different stages of female bud (1, 2, 5, and 10-mm buds were divided into 5-mm bud, 5-mm ovary, 10-mm bud, and 10-mm ovary, respectively) and spatial expression patterns of petal, stamen, stigma, mesocarp, and exocarp in the female flower before antheses according to qRT-PCR. Error bars represent the SD from three biological replicates. Different letters (a–g) indicate significant differences (*P* < 0.05) of *CsERF31* and *M* in different flower organs. **(B)** Schematic diagram of sex expression of the flowers from the 5th to 25th nodes at the main stems in G12 and M12 treated with ethylene-related chemical (C_2_H_4_, ethephon; AVG, aminoethoxyvinyl glycine; AgNO_3_) and deionized water (Control), respectively. The statistical data of sexual types with the five biological replicates are shown in **Supplementary Figure S2**. The flowers observed in lateral branches were not recorded. **(C)** Expression patterns of *M* and *CsERF31* in shoot apices of the chemical treatments and control lines. Error bars represent the SD from the five biological replicates, and different letters (a–c) indicate significant differences (*P* < 0.05) of *CsERF31* and *M* in different treatments.

To further explore the role of *CsERFs* in sex expression, we verified the expression patterns of seven *ERFs* of DEGs by qRT-PCR. Only *CsERF31* showed an expression pattern similar to that of *M* (**Figure 3**). Furthermore, we tested the spatiotemporal expression of *CsERF31* and *M* in G12 flowers. The expression levels of *CsERF31* and *M* were very low in different organs of female flowers that were ready to blossom. However, *CsERF31* and *M* were expressed at a high level in the 1- and 2-mm buds (i.e., at ∼stages 6 and 10, respectively), while the expression levels were reduced in both the 5- and 10-mm buds/ovaries (**Figure 4A**).

The ethylene-related chemical treatments modulated the sexual types of the cucumber flower buds. The treatment of the shoot apices with ethephon generated female flowers at low nodes in M12, whereas no effect was observed in G12. The treatments with AVG and AgNO_3_ generated male flowers in G12, whereas no effect was observed in M12 (**Figure 4B** and **Supplementary Figure S2**). Accordingly, the expression levels of *CsERF31* and *M* were significantly upregulated in ethephon-treated G12 and M12. However, the expression levels were downregulated only in G12 treated with AVG or AgNO_3_ (**Figure 4C**). In conclusion, *CsERF31* and *M* showed a highly synchronous expression pattern among a variety of samples.

### *CsERF31* Bind the ERE-Box in the *M* Promoter and Activate Its Expression

**FIGURE 5:**
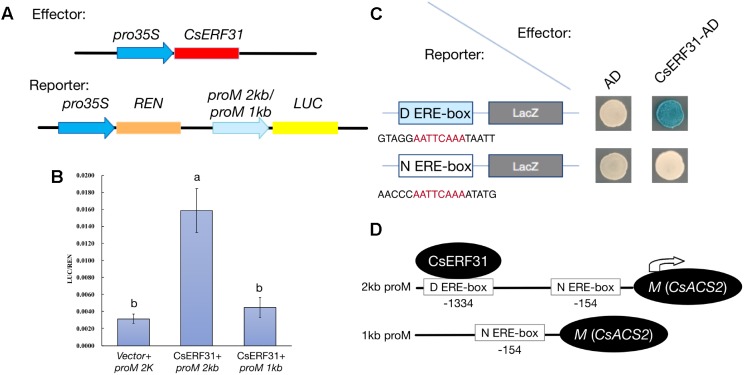
Effects of *CsERF31* on the transcriptional activity of *M* promoter. **(A)** Schematic diagram of the constructs using Dual-LUC analysis in a tobacco transient expression system. Effector constructs contained the CaMV 35S promoter fused to the transcription factors *CsERF31*. Reporter constructs contained 2 or 1 kb of *M* promoter, upstream of the translation initiation sites, fused to the *LUC* reporter gene. REN was used as the internal control. **(B)** Effects of *CsERF31* on the activity of 2 kb (two ERE-box in it) or 1 kb (only the near ERE-box in it) of *M* promoter. Error bars represent the SD from four biological replicates, and different letters (a, b) indicate significant differences (*P* < 0.05) of the transcriptional activation activity with different promoter regions. **(C)** Yeast one-hybrid assay of interaction between *CsERF31* and the distant ERE-box (D ERE-box) or the near ERE-box (N ERE-box). **(D)** Schematic diagram of *CsERF31* binding the D ERE-box in *M* promoter to activate *M* transcription.

To obtain additional evidence of the activation effect of *CsERF31* on the *M* expression, we conducted heterologous transient expression experiments in tobacco. The −2 and −1 kb sequences upstream of the translation start site of *M* were fused to sequence encoding LUC to construct the reporters *proM2kb:LUC* and *proM1kb:LUC*, respectively (**Figure 5A**). In addition, a construct containing the coding sequence of *CsERF31* downstream of the constitutive 35S promoter was generated. The two constructs were simultaneously introduced into tobacco leaves. Compared to the control (empty vector + *proM2kb:LUC*), the transcriptional activation activity (LUC/REN) of *proM2kb:LUC*, but not *proM1kb:LUC*, was significantly promoted by *CsERF31* (**Figure 5B**). We further investigated whether *CsERF31* directly activated the *M* expression. The yeast one-hybrid assay revealed that *CsERF31* was bound to the distant ERE-box (D ERE-box, −1334) but not the near ERE-box (N ERE-box, −154) of the *M* promoter (**Figure 5C**). In conclusion, *CsERF31* could bind to the ERE-box in −1334 upstream of *M* to activate its transcription but could not bind to the near ERE-box −154 upstream of *M* (**Figure 5D**). In addition, the ORFs sequence of *CsERF31* and the 2-kb promotor sequence of *M* are consistent among the NILs (unpublished data).

## Discussion

In general, the wild and semi-wild cucumber varieties (e.g., *hardwickii* and *xishuangbanna*) are monoecious (*ffMMAA*), indicating that the sexual types of flower buds in the shoot apices are indeterminate. However, a different result is observed if the genotype is *FFMMAA* or *FFmmAA* because the sex of the buds could be predetermined at the whole plant level. Previous study indicates that the sex expression is directly related to ethylene release rate in different genotype cucumbers (e.g., *FFMMAA*, *ffMMAA*, and *FFmmAA*) (Cui et al., 2016). Besides, in our previous study, the ethylene release rate and *M* expression both show the gradually decreasing trend in *FFMMAA*, *FFmmAA*, and *ffMMAA* cucumber lines (Li et al., 2012). Thus, we propose that NILs with three genotypes could be ideal materials to investigate the process of female flower differentiation, considering both *F* and *M*. The main purpose of this study was to provide valuable data and clues for exploring the molecular mechanism of cucumber unisexual development, particularly female flower differentiation. In this study, we identified that *CsERF31* acts as a regulator of *M*. Although the function of *CsERF31* requires further investigation, our data should be further explored to identify the relevant genes and their networks in the process of female flower differentiation in the cucumber.

### The Unisexual Flower in Cucumber May Be Characterized by the Special Expression Patterns of B- and C-Class Genes

The identification of ABC genes that determine the floral organ identities (Coen and Meyerowitz, 1991) was considered a breakthrough in the understanding of sex differentiation (Bai and Xu, 2012). In this study, as **Figure 2** and **Table 1** depict, the expression of the B-class genes *CsPI* (putative ortholog of *PISTILLATA* in *Arabidopsis*) and *CsAP3* was markedly suppressed in G12, which was consistent with our predictions, suggesting that the B-class genes are functionally conservative in cucumber. Moreover, the *CsSUP* (putative ortholog of *SUPERMAN*/*FLO10* in *Arabidopsis*) transcripts were only increased in G12, showing an expression pattern opposite to that of *CsPI* and *CsAP3*. The *SUP/FLO10* have been well studied and are considered cadastral genes that act indirectly to prevent B-class genes from acting in the gynoecial whorl in *Arabidopsis* (Schultz et al., 1991; Bowman et al., 1992; Gaiser et al., 1995; Jacobsen and Meyerowitz, 1997). Thus, the reduced transcripts of *CsPI* and *CsAP3* might be caused by the upregulation of *CsSUP* in G12. In addition, *CsWUS* was recently reported to play a key role in promoting flower development by activating *CsAP3* and *CUM1* (Zhao et al., 2017), which showed 10-fold higher expression in M12 and H34 when compared to that in G12. Therefore, we speculated that *CsWUS* and *CsSUP* act as regulators that stimulate and suppress the transcription of *CsAP3*, respectively, in different genotypes to control sex expression. Furthermore, previous studies reveal that *CsAP3* also performs unique functions in regulating cucumber flower development by activating the *CsETR1* expression (Sun et al., 2016), thus establishing a direct connection between ethylene and B-class genes.

**Table 1:** Information of the genes present in the predicted protein interaction network.

Name	Cucumber ID	Gene information	Arabidopsis best hit	Foldchange (G12/M12)	Foldchange (G12/H34)
ACS7	*Csa1G580750*	1-aminocyclopropane-1-carboxylate synthase	*AT4G26200*	∞	2.65
CKX3	*Csa1G588560*	Cytokinin oxidase/dehydrogenase	*AT5G56970*	2.08	1.10
EFE	*Csa6G421630*	1-aminocyclopropane-1-carboxylate oxidase	*AT1G05010*	8.58	4.69
JAZ8	*Csa6G523460*	Jasmonate ZIM domain protein f	*AT1G30135*	2.12	1.21
JAZ1	*Csa1G597690*	JAZ1 (JASMONATE-ZIM-DOMAIN PROTEIN 1)	*AT1G19180*	2.32	1.19
ERF1	*Csa3G135120*	Ethylene-responsive transcription factor 1B	*AT3G23240*	∞	13.32
MDP	*Csa1G471470*	Malate dehydrogenase	*AT5G58330*	3.71	3.04
BFT	*Csa3G180440*	Terminal flower 1b	*AT5G62040*	36.41	18.20
AP1	*Csa1G051580*	MADS-box transcription factor	*AT1G69120*	41.84	28.87
SUP	*Csa3G141870*	Putative SUPERMAN-like transcription factor	*AT3G23130*	36.62	1.72
CRC	*Csa5G606780*	Protein CRABS CLAW, putative	*AT1G69180*	62.58	3.74
SHP2	*Csa1G467100*	CAG2, AG-like C-class floral gene	*AT2G42830*	41.84	28.87
WUS	Csa6G505860	WUSCHEL (WUS)	*AT2G17950*	0.09	0.09
SPL	*Csa3G850670*	SPOROCYTELESS (SPL)	*AT4G27330*	0.04	0.03
PI	*Csa4G358770*	PISTILLATA (PI)	*AT5G20240*	0.31	0.34
AP3	*Csa3G865440*	APETALA 3 (AP3)	*AT3G54340*	0.44	0.49
AGL2	*Csa1G039900*	SEPALLATA1 (SEP1)	*AT5G15800*	0.27	0.30
AIL6	*Csa3G114480*	AINTEGUMENTA-like 6	*AT5G10510*	0.49	0.67
SEP4	*Csa6G367080*	SEPALLATA 4 (SEP4)	*AT2G03710*	0.42	0.65

Interestingly, the transcripts of a C-class AG homolog, *CAG2*, which did not appear to be modulated by gibberellin or ethylene in the previous studies (Kahana et al., 1999), presented a gradient reduction in G12, H34, and M12. Previous studies have also demonstrated that *CAG2* was specifically expressed in the ovary (Kahana et al., 1999), which is an organ that develops relatively late in the female flower. Hence, we speculated that *CAG2* may not directly respond to ethylene but is likely to play an essential role in ovary development after the female flower is initiated. The insufficient expression level of *CAG2* in H34 helps explain the reason for its short fruit. Altogether, we suggest that both B- and C-class genes are involved in sex expression and are selectively suppressed or promoted by endogenous ethylene to form male or female flowers, respectively. However, evidence supporting the causality between ethylene and B- and C-class genes must be provided in further studies.

### Investigation of the Positive Feedback Regulation of *M* Increases Our Understanding of Cucumber Unisexual Flower Development and Evolution

The feedback regulation of *M* renders the “one-hormone hypothesis” more powerful in explaining the sex expression of cucumbers. Meanwhile, the amplified ethylene signal triggered further questions regarding why and how this process evolved. To study the positive feedback of *M*, we performed a co-expression trend analysis to screen for *M*-related genes among the DEGs. Consequently, *CsERF31* was identified with the characteristics of co-expression (**Figure 4A**), co-responding (**Figure 4C**), and directly interacting (**Figure 5**) with *M*. Notably, *CsERF31* and *M* were specifically expressed in 1- to 2-mm flower buds and faded in 5- to 10-mm flower buds in G12 (**Figure 4A**). Thus, we speculate that the expression of *CsERF31* and *M* may be activated at the initial stages of flower buds and then immediately suppressed via a cucumber unique mechanism. The highly synchronous expression pattern implied a strong correlation between *CsERF31* and *M*, which led us to verify the direct transcriptional activation of *M* by *CsERF31*. Interestingly, *CsERF31* could activate the *M* expression only by binding to the distant, not the near, ERE-box from the *M* ORF (**Figure 5**). Thus, other ERF family genes are likely to bind to the near ERE-box to regulate *M*. In this study, *CsERF31* and *M* presented different expression patterns with six other ERF genes (**Figure 3**). Thus, it is likely that the other six ERFs are not involved in the positive feedback regulation or serve as negative regulators in ethylene signaling (Han et al., 2016). However, the regulation mechanisms of *M* remain obscure and should be explored in further studies.

**FIGURE 6:**
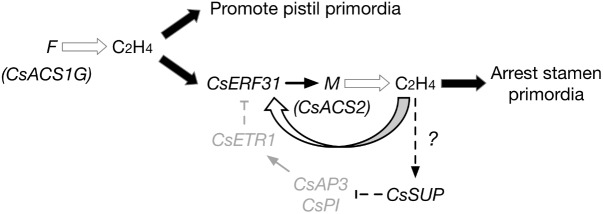
Model of *F* and *M* functions during the differentiation of female flowers in gynoecious cucumber (*FFMMAA*). *F* produces ethylene, promotes pistil primordia development. *CsERF31* responds to the ethylene signal and activates *M*. *M* begins its positive feedback activating expression via *CsERF31*. The continuous accumulation of ethylene downregulates the B-class genes, *CsAP3*, and *CsPI*, by stimulating *CsSUP* in an unknown way. Thus, expression of *CsETR1* is suppressed and fails to downregulate *CsERF31*. This process constantly arrests the development of stamen primordia, resulting in the initiation of female flowers.

Combining the understanding from previous data regarding *F* and *M*, the following extended model of sex differentiation in cucumber is proposed (**Figure 6**):

(1) In gynoecious cucumber (*FFMMAA*), according to the model, the structural variation in the *F* locus (Zhang et al., 2015) causes a constitutive expression of *M* in all flower buds and produces female flowers only at the whole plant level. However, the model may not be applicable to monoecious cucumber (*ffMMAA*) because no evidence exists that *M* is activated by *F* (or ethylene produced by F) in the predetermined female buds in consideration of *A*, which is epistatic in melon (Boualem et al., 2015). In conclusion, we suppose that the *M* expression is activated by ethylene signaling produced by *F* (*CsACS1G*) in *FFMMAA* genotypes and by *f* (*CsACS1*) or *A* in *ffMMAA* genotypes.
(2) Compared with the mechanism of ethylene accumulation, the detailed mechanism explaining the different fates of pistil and stamen primordia is more intriguing. According to the aforementioned DEGs, we hypothesize that *SUP*, *AP3*, and *PI* play the same key roles in both cucumber and *Arabidopsis*. Notably, *CsSUP* may be regulated via an unknown mechanism related to ethylene. In addition, the downregulation of *CsETR1* triggers the aberrant development of stamen in female flowers (Wang et al., 2010), suggesting that *CsETR1* may act as a negative regulator in the *CsERF31*-involved ethylene signaling pathway.
(3) If an “ethylene-*CsERF31*-*M*-ethylene” positive feedback can be activated in female buds, then the question arises as to how this process could be accurately controlled. Furthermore, if an ethylene negative or fine-tuning regulatory mechanism exists in female buds, this mechanism should be a breakthrough to address the long-standing question of cucumber sex differentiation.

### Transcriptome Analysis Suggests That the Female Reproductive Organs May Be Regulated via Ethylene-Auxin Crosstalk

Successful fertilization in plants requires the properly coordinated development of the female reproductive organs. In this study, as the DEGs depict in **Supplementary Tables S2, S3**, the predicted cucumber *HECATE* (*HEC*) (*Csa2G285890*) showed 45-fold higher expression in G12 than in M12 and H34. The *HEC* encodes a basic-helix-loop-helix (bHLH) protein that can dimerize with SPTTULA (SPT), which is involved in the auxin-mediated control of gynoecium patterning in *Arabidopsis* (Gremski et al., 2007). In addition, previous studies have demonstrated that SPT coordinates with additional bHLH members, such as CRABS CLAW (CRC), to regulate carpel and fruit development by regulating auxin distribution in *Arabidopsis* (Alvarez and Smyth, 1999; Heisler et al., 2001; Girin et al., 2011). Unsurprisingly, the putative cucumber *CRC* showed a 3.8-fold increase in G12 compared to that in H34 (**Figure 2D**). These similar expression trends suggest that the regulatory mechanism of floral organogenesis may be conserved between cucumber and *Arabidopsis*. Thus, we speculate that the crosstalk network between ethylene and auxin may be involved in modulating the floral organogenesis in cucumbers.

The Cucumber fruit is a type of fleshy fruit that is harvested while still immature. The ovaries and fruits of most cucumber varieties with an *M* locus are elongated, whereas those of hermaphroditic plants with *m* alleles are oval/round shaped (Tan et al., 2015). A recent study has shown that the AUX/IAA genes *SlIAA29* can respond to ethylene signaling, and this study revealed a strong correlation between the auxin- and ethylene-related genes, suggesting a significant crosstalk between auxin and ethylene during tomato ripening (Zhang, 2017). According to our data, the transcripts of the indole-3-acetic acid (IAA)-inducible gene *CsIAA29* (*Csa3G877650*) were upregulated in G12. Furthermore, the putative cucumber histidine phosphotransfer protein (AHP) (*Csa2G285890*), which is a positive regulator of cytokinin signaling in *Arabidopsis* (Hutchison et al., 2006), displayed a 3.3-fold higher expression in G12. Besides, a previous study has shown that AHP can interact with the ethylene receptor ETR1 (Scharein et al., 2008), suggesting that the ethylene-cytokinin interaction is likely to exist in cucumber fruit development. Similarly, the putative cucumber *FRUITFULL* (*FUL*) (*Csa1G039910*), which is a MADS-box gene regulating cell differentiation during fruit development in *Arabidopsis* (Gu et al., 1998) and fruit ripening in tomato (Fujisawa et al., 2014), showed a twofold reduction in H34. Notably, the aforementioned genes, *CsIAA29*, *CsCRC*, and *CsFUL*, displayed similar expression trends in a previous study investigating the fruit length in cucumbers (Jiang et al., 2015), thus suggesting that these genes may play pivotal roles in early fruit development. Interestingly, *CsSPL*, which encodes a protein that directly interacts with CsWUS (WUSCHEL) and positively regulates *CsWUS* and *CsARF3* (*AUXIN RESPONSE FACTOR*3) expression (Liu et al., 2018), showed a 4.4-fold higher expression in M12 and H34. Thus, *CsSPL* not only regulates sex organ development by interacting with auxin signaling, as recently reported, but may also participate in the process of sex differentiation in cucumbers.

### Exploring Gene Function and Regulatory Networks of Cucumber Sex Differentiation in Model Plants, Are Equally Important

Previous studies have demonstrated that the organ-specific downregulation of the ethylene receptor gene (*CsETR1*) or upregulation of the ethylene synthesis gene (*CsACO2*) in transgenic *Arabidopsis* plants can generate female flowers (Duan et al., 2008; Wang et al., 2010). Subsequent studies have demonstrated that *CsACO2* is essential for female flower initiation and is regulated by *CsWIP1* in cucumbers (Chen et al., 2016). Previous studies have also shown that *At3G23240* (*AtERF1*) is upregulated in *CsETR1* downregulated transgenic *Arabidopsis*, and “cucumber homolog of *At3G23240*” has a highly specific expression pattern at stage 6 in female flowers (Wang et al., 2010). In our study, *CsERF31* was also a “cucumber homolog of *At3G23240*,” and its expression was consistently increased significantly at stage 6 in the female flowers (∼1 mm flower buds of G12) (**Figure 4A**) to promote the transcription of *M* (**Figure 6**). Additionally, previous studies investigating *CsAP3* have shown a conservative function by rescuing the *Arabidopsis* *ap3* mutant, but this is a novel function in cucumber by activating *CsETR1* transcription (Sun et al., 2016). Besides, *CUM1* was identified in petunia (*Petunia hybrida*) with the function of inducing reproductive organ fate (Kater et al., 1998), and the function is supported by *in situ* hybridization in a recent study (Ran et al., 2018). Overall, these findings in cucumber and *Arabidopsis* or other species complement and verify each other and have inspired the understanding of sex differentiation in plants in a broader perspective.

Our main purpose for studying cucumber sexual expression was to understand the role played by ethylene in this process, particularly the characteristics that distinguish the ethylene regulatory mechanism in cucumbers from that in other plants. Therefore, we consider that the functional studies investigating cucumber sex differentiation-related genes in well-studied plants, such as *Arabidopsis*, are efficient and provide practical approaches to discovering the regulatory mechanism from a multispecies evolutionary perspective in angiosperm. In conclusion, cucumber sex differentiation should be studied and understood as an evolutionary event more than traditional floral development.

## Author Contributions

JSP and RC designed the experiments. JP and HW performed the experiments. GW, HL, and JP analyzed the data. JP wrote the paper along with HD. HH planted the cucumber plants.

## Conflict of Interest Statement

The authors declare that the research was conducted in the absence of any commercial or financial relationships that could be construed as a potential conflict of interest.

## Footnotes

1 http://cucurbitgenomics.org

2 http://www.omicshare.com/tools

3 https://string-db.org
