# Supplementary material for: Differential Gene Expression Caused by the F and M Loci Provides Insight Into Ethylene-Mediated Female Flower Differentiation in Cucumber
Source: Front Plant Sci. 2018 Aug 14;9:1091. doi: 10.3389/fpls.2018.01091 (PMC6102477; doi:10.3389/fpls.2018.01091)
Supplement: Supplementary file 2 [file Image_2.PDF]

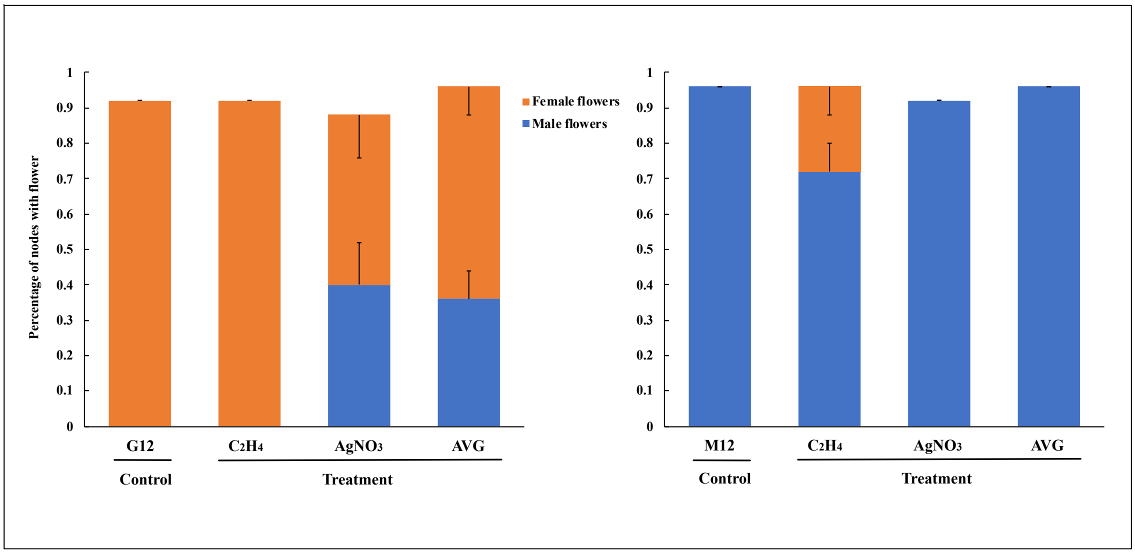

Supplementary Figure S2 | Effects of chemical treatment on sex expression in cucumber plants. The percentage of the nodes with female or male flowers of the Control and Treatment lines. The sex of the first flowered bud on each node was recoded up to the 25th node on the main stems. Error bars indicate the value of the least significant individual in sex modulating from five independent plants.
