# Supplementary material for: Differential Gene Expression Caused by the F and M Loci Provides Insight Into Ethylene-Mediated Female Flower Differentiation in Cucumber
Source: Front Plant Sci. 2018 Aug 14;9:1091. doi: 10.3389/fpls.2018.01091 (PMC6102477; doi:10.3389/fpls.2018.01091)
Supplement: Supplementary file 1 [file Image_1.PDF]

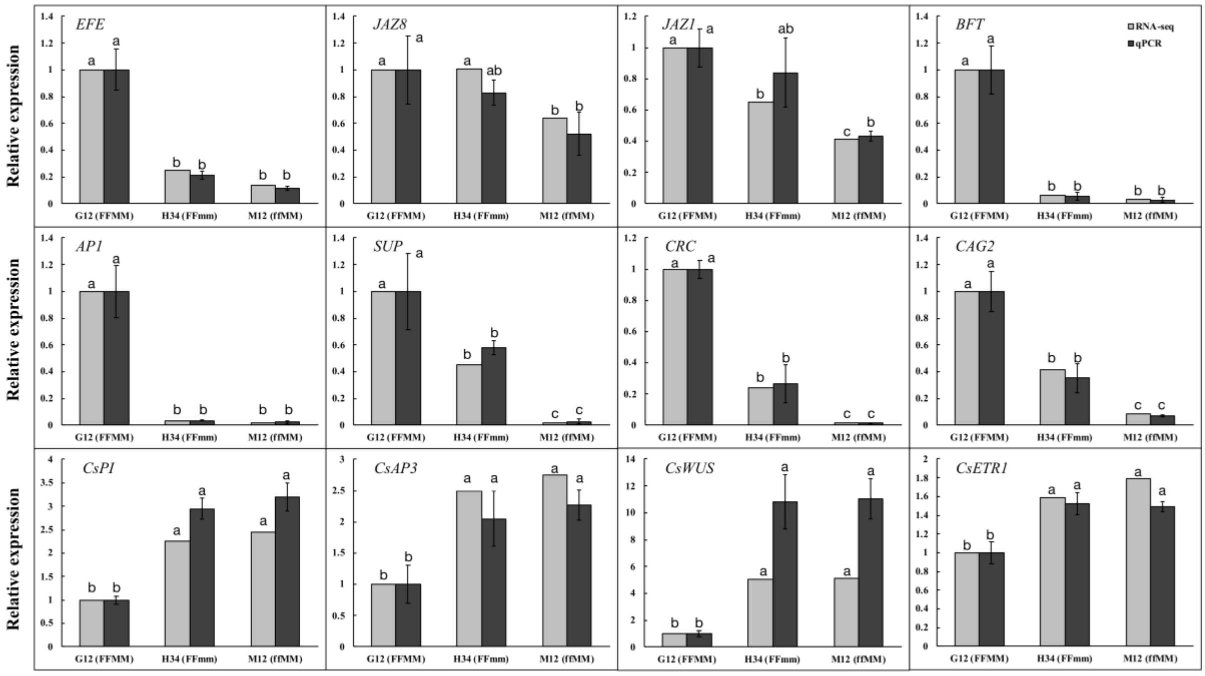

Supplementary Figure S1 | qRT-PCR validation of DEGs identified by RNA-Seq. A total of 12 genes selected from DEGs of the two sets of transcriptome comparisons (G12 vs. M12 and G12 vs. H34) for qRT-PCR confirmation. The cucumber *Actin* was used as an internal control, and these experiments were repeated with three biological samples. Error bars indicate the standard errors. Different letters (a, b, c) indicate significant expression differences ($P < 0.05$) of the 12 genes in G12, H34 and M12.
